# *Bembidion ambiguum* (Coleoptera: Carabidae) is established in California

**DOI:** 10.3897/BDJ.6.e30763

**Published:** 2018-12-04

**Authors:** David R. Maddison, Kipling Will, Sarah Crews, James LaBonte

**Affiliations:** 1 Oregon State University, Corvallis, United States of America Oregon State University Corvallis United States of America; 2 University of California, Berkeley, Berkeley, CA, United States of America University of California, Berkeley Berkeley, CA United States of America; 3 California Academy of Sciences, San Francisco, United States of America California Academy of Sciences San Francisco United States of America; 4 Oregon Department of Agriculture, Salem, United States of America Oregon Department of Agriculture Salem United States of America

**Keywords:** Carabidae, Bembidiini, introduced species, Mediterranean

## Abstract

**Background:**

The ground beetle Bembidion (Neja) ambiguum Dejean is native to Europe and north Africa, in the Mediterranean region.

**New information:**

We report it from North America for the first time, from five localities around San Francisco Bay, California. The earliest record is from 2012.

## Introduction

Of the approximately 2,400 carabid species known from America north of Mexico, 64 are non-native, with the majority of these being native to Europe (Bousquet 2012). Within the large genus *Bembidion* Latreille, eight of the approximately 260 described species in North America were accidentally introduced from Europe (Bousquet 2012). The most recent of these was *Bembidion femoratum* Sturm, which appeared in North America by 1967 (Bousquet 1992). With the exception of the now-widespread *Bembidion tetracolum* Say, these eight introduced *Bembidion* species are now restricted to northeastern North America and the Pacific Northwest (Bousquet 2012).

The discovery of a specimen of a *Bembidion* in Oakland, California, by one of the authors (SC) led to the realization that a ninth European *Bembidion* species had made its way to North America. This species, *Bembidion ambiguum* Dejean, is the first representative of the subgenus Neja Motschulsky to be found in North America, and the first introduced *Bembidion* species natively restricted to Europe and North Africa's Mediterranean region.

## Materials and methods

Basic methods for studying adult structures, and terms used, are given in Maddison (1993). Body length was measured from the front of the labrum to the elytral apex. Genitalia have been mounted in Euparal between two small coverslips attached to archival-quality heavyweight watercolor paper.

Photographs of body parts were taken with a Leica Z6 Apo lens and DMC4500 camera, and of male genitalia with a Leica DM5500B compound microscope and DMC425C camera. A stack of images from different focal positions was then merged using the PMax procedure in Zerene Systems’s Zerene Stacker; the final images thus potentially have some artifacts caused by the merging algorithm.

Specimens examined are housed in the following collections: Essig Museum Entomological Collection, University of California, Berkeley (EMEC), James R. LaBonte collection, Dallas, Oregon (JRLC) and Oregon State Arthropod Collection, Oregon State University, Corvallis (OSAC).

## Taxon treatments

### Bembidion (Neja) ambiguum

Dejean, 1831

#### Materials

- **Type status:** Other material. **Occurrence:** catalogNumber: DRMaddison DNA5191; recordedBy: David R. Maddison; individualCount: 1; sex: male; lifeStage: adult; occurrenceID: urn:catalog:OSAC:DRMaddison:DNA5191; **Taxon:** taxonID: urn:lsid:catalogueoflife.org:taxon:1d9f293d-ac8f-11e3-805d-020044200006:col20150401; scientificName: Bembidion ambiguum; class: Insecta; order: Coleoptera; family: Carabidae; genus: Bembidion; subgenus: Neja; specificEpithet: ambiguum; **Location:** country: USA; stateProvince: California; county: Alameda; locality: Oakland; decimalLatitude: 37.812; decimalLongitude: -122.2717; georeferenceProtocol: label; **Identification:** identifiedBy: David R. Maddison; dateIdentified: 2017; **Event:** eventDate: 04/23/2017; **Record Level:** institutionCode: OSAC; basisOfRecord: PreservedSpecimen
- **Type status:** Other material. **Occurrence:** catalogNumber: EMEC346364; recordedBy: David R. Maddison; individualCount: 1; sex: female; lifeStage: adult; occurrenceID: urn:catalog:EMEC:EMEC:346364; **Taxon:** taxonID: urn:lsid:catalogueoflife.org:taxon:1d9f293d-ac8f-11e3-805d-020044200006:col20150401; scientificName: Bembidion ambiguum; class: Insecta; order: Coleoptera; family: Carabidae; genus: Bembidion; subgenus: Neja; specificEpithet: ambiguum; **Location:** country: USA; stateProvince: California; county: Alameda; locality: Oakland; decimalLatitude: 37.812; decimalLongitude: -122.2717; georeferenceProtocol: label; **Identification:** identifiedBy: Kipling Will; dateIdentified: 2017; **Event:** eventDate: 11/19/2017; **Record Level:** institutionCode: EMEC; basisOfRecord: PreservedSpecimen
- **Type status:** Other material. **Occurrence:** catalogNumber: EMEC346365; recordedBy: David R. Maddison; individualCount: 1; sex: male; lifeStage: adult; occurrenceID: urn:catalog:EMEC:EMEC:346365; **Taxon:** taxonID: urn:lsid:catalogueoflife.org:taxon:1d9f293d-ac8f-11e3-805d-020044200006:col20150401; scientificName: Bembidion ambiguum; class: Insecta; order: Coleoptera; family: Carabidae; genus: Bembidion; subgenus: Neja; specificEpithet: ambiguum; **Location:** country: USA; stateProvince: California; county: Alameda; locality: Oakland; decimalLatitude: 37.812; decimalLongitude: -122.2717; georeferenceProtocol: label; **Identification:** identifiedBy: Kipling Will; dateIdentified: 2017; **Event:** eventDate: 12/02/2017; **Record Level:** institutionCode: EMEC; basisOfRecord: PreservedSpecimen
- **Type status:** Other material. **Occurrence:** catalogNumber: EMEC346362; recordedBy: David R. Maddison; individualCount: 1; sex: male; lifeStage: adult; occurrenceID: urn:catalog:EMEC:EMEC:346362; **Taxon:** taxonID: urn:lsid:catalogueoflife.org:taxon:1d9f293d-ac8f-11e3-805d-020044200006:col20150401; scientificName: Bembidion ambiguum; class: Insecta; order: Coleoptera; family: Carabidae; genus: Bembidion; subgenus: Neja; specificEpithet: ambiguum; **Location:** country: USA; stateProvince: California; county: Santa Clara; locality: Santa Clara, Forge Garden and Santa Clara University; decimalLatitude: 37.3523; decimalLongitude: -121.9394; coordinateUncertaintyInMeters: Santa Clara, Forge Garden and Santa Clara University; georeferencedBy: David R. Maddison; georeferenceProtocol: Found Forge Garden on Google Maps; georeferenceSources: http://maps.google.com; **Identification:** identifiedBy: Kipling Will; dateIdentified: 2018; **Event:** eventDate: 05/20/2013; **Record Level:** institutionCode: EMEC; basisOfRecord: PreservedSpecimen
- **Type status:** Other material. **Occurrence:** catalogNumber: EMEC346363; recordedBy: David R. Maddison; individualCount: 1; sex: female; lifeStage: adult; occurrenceID: urn:catalog:EMEC:EMEC:346363; **Taxon:** taxonID: urn:lsid:catalogueoflife.org:taxon:1d9f293d-ac8f-11e3-805d-020044200006:col20150401; scientificName: Bembidion ambiguum; class: Insecta; order: Coleoptera; family: Carabidae; genus: Bembidion; subgenus: Neja; specificEpithet: ambiguum; **Location:** country: USA; stateProvince: California; county: Santa Clara; locality: Santa Clara, Forge Garden and Santa Clara University; decimalLatitude: 37.3523; decimalLongitude: -121.9394; coordinateUncertaintyInMeters: Santa Clara, Forge Garden and Santa Clara University; georeferencedBy: David R. Maddison; georeferenceProtocol: Found Forge Garden on Google Maps; georeferenceSources: http://maps.google.com; **Identification:** identifiedBy: Kipling Will; dateIdentified: 2018; **Event:** eventDate: 05/20/2013; **Record Level:** institutionCode: EMEC; basisOfRecord: PreservedSpecimen
- **Type status:** Other material. **Occurrence:** recordedBy: David R. Maddison; individualCount: 1; sex: female; lifeStage: adult; occurrenceID: urn:catalog:JRLC:Carabidae:Bembidion.ambiguum.1; **Taxon:** taxonID: urn:lsid:catalogueoflife.org:taxon:1d9f293d-ac8f-11e3-805d-020044200006:col20150401; scientificName: Bembidion ambiguum; class: Insecta; order: Coleoptera; family: Carabidae; genus: Bembidion; subgenus: Neja; specificEpithet: ambiguum; **Location:** country: USA; stateProvince: California; county: Santa Clara; locality: Palo Alto, Stanford University Campus; decimalLatitude: 37.4224; decimalLongitude: -122.18124; coordinateUncertaintyInMeters: Palo Alto, Stanford University Campus; georeferenceProtocol: label; **Identification:** identifiedBy: James R. LaBonte; dateIdentified: 2018; **Event:** eventDate: 11/12/2012; **Record Level:** institutionCode: JRLC; basisOfRecord: PreservedSpecimen
- **Type status:** Other material. **Occurrence:** recordedBy: David R. Maddison; individualCount: 1; sex: female; lifeStage: adult; occurrenceID: urn:catalog:JRLC:Carabidae:Bembidion.ambiguum.2; **Taxon:** taxonID: urn:lsid:catalogueoflife.org:taxon:1d9f293d-ac8f-11e3-805d-020044200006:col20150401; scientificName: Bembidion ambiguum; class: Insecta; order: Coleoptera; family: Carabidae; genus: Bembidion; subgenus: Neja; specificEpithet: ambiguum; **Location:** country: USA; stateProvince: California; county: Santa Clara; locality: Gilroy, Thousand Trails; decimalLatitude: 37.057; decimalLongitude: -121.668; coordinateUncertaintyInMeters: Gilroy, Thousand Trails; georeferencedBy: David R. Maddison; georeferenceProtocol: Found Thousand Trails campsite on Google Maps; georeferenceSources: http://maps.google.com; **Identification:** identifiedBy: James R. LaBonte; dateIdentified: 2018; **Event:** eventDate: 11/04/2015; **Record Level:** institutionCode: JRLC; basisOfRecord: PreservedSpecimen
- **Type status:** Other material. **Occurrence:** recordedBy: David R. Maddison; individualCount: 1; sex: male; lifeStage: adult; occurrenceID: urn:catalog:JRLC:Carabidae:Bembidion.ambiguum.3; **Taxon:** taxonID: urn:lsid:catalogueoflife.org:taxon:1d9f293d-ac8f-11e3-805d-020044200006:col20150401; scientificName: Bembidion ambiguum; class: Insecta; order: Coleoptera; family: Carabidae; genus: Bembidion; subgenus: Neja; specificEpithet: ambiguum; **Location:** country: USA; stateProvince: California; county: Santa Clara; locality: San Jose, 1890 Dobbin Drive; decimalLatitude: 37.367; decimalLongitude: -121.8636; coordinateUncertaintyInMeters: San Jose, 1890 Dobbin Drive; georeferencedBy: David R. Maddison; georeferenceProtocol: Found 1890 Dobbin Drive, Santa Clara, on Google Maps; georeferenceSources: http://maps.google.com; **Identification:** identifiedBy: James R. LaBonte; dateIdentified: 2018; **Event:** eventDate: 12/21/2016; **Record Level:** institutionCode: JRLC; basisOfRecord: PreservedSpecimen

#### Diagnosis

**Figure 1.**
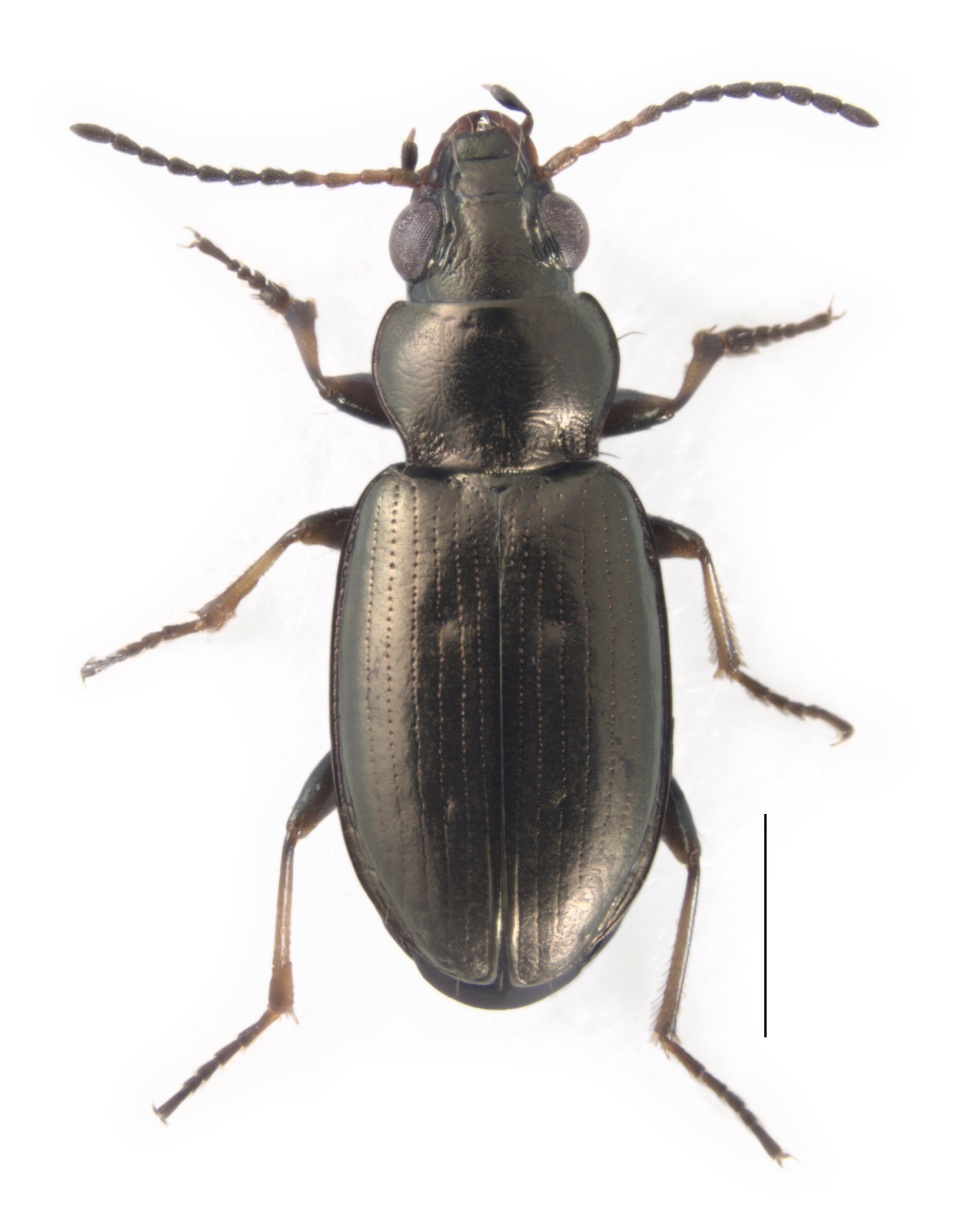
*Bembidion ambiguum* from Oakland, California. Scale bar is 1.0 mm.

**Figure 2.**
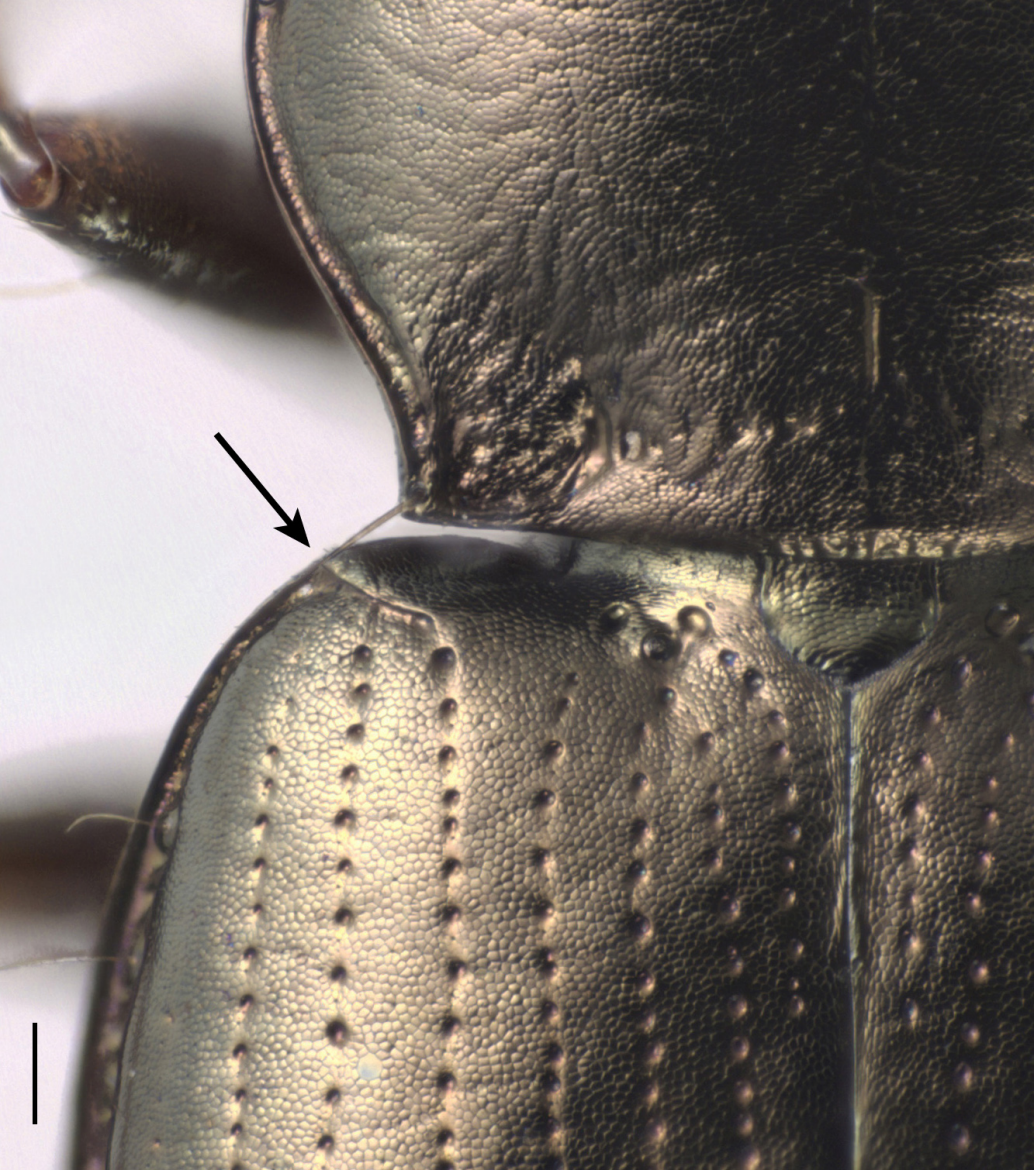
*Bembidion ambiguum*, closeup of shoulder of elytron, showing the angled margin. The arrow points to the vertex of the angle. Scale bar is 0.1 mm.

**Figure 3.**
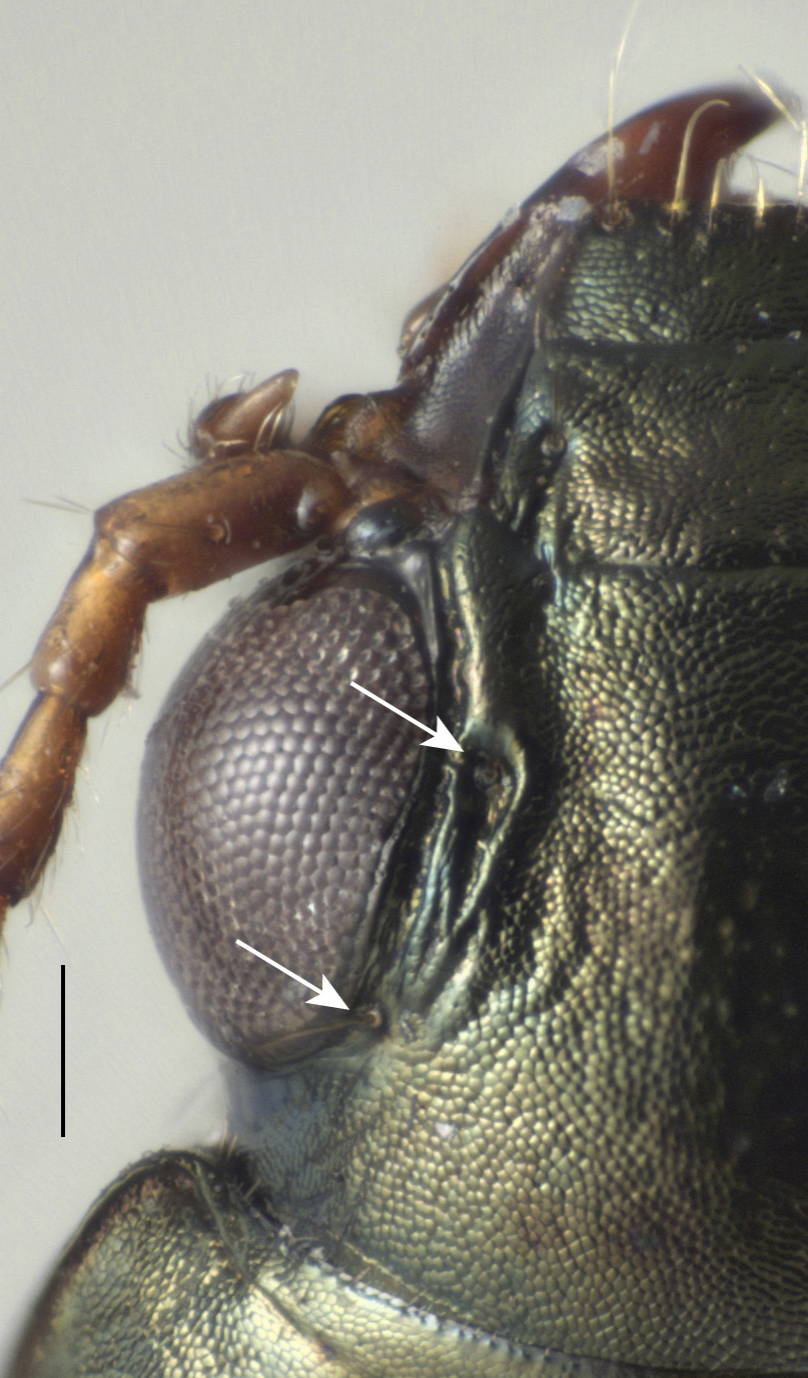
*Bembidion ambiguum*, left side of head. The base of the two supraorbital setae are indicated by the arrows. Scale bar is 0.1 mm.

Distinctive within the North American fauna in having the following combination of characteristics: small (3.5–4.2 mm in length), metallic coloration, with surface dull because of well-impressed, isodiametric sculpticells (Fig. 1). Pronotum slightly constricted posteriorly (Fig. 2); posterior margin slightly narrower than anterior margin. Elytron with humeral margin forming a sharp angle (Fig. 2), with lateral groove reaching to near the base of the fourth stria; two discal setae contained in small foveae. In addition, the anterior supraorbital seta is in a furrow that extends back toward the posterior supraorbital setae (Fig. 3); this furrow is separated from the central portion of the head by a carina. The internal sclerites of the male genitalia are also distinctive within the North American fauna (Fig. 4).

#### Distribution

**Figure 5.**
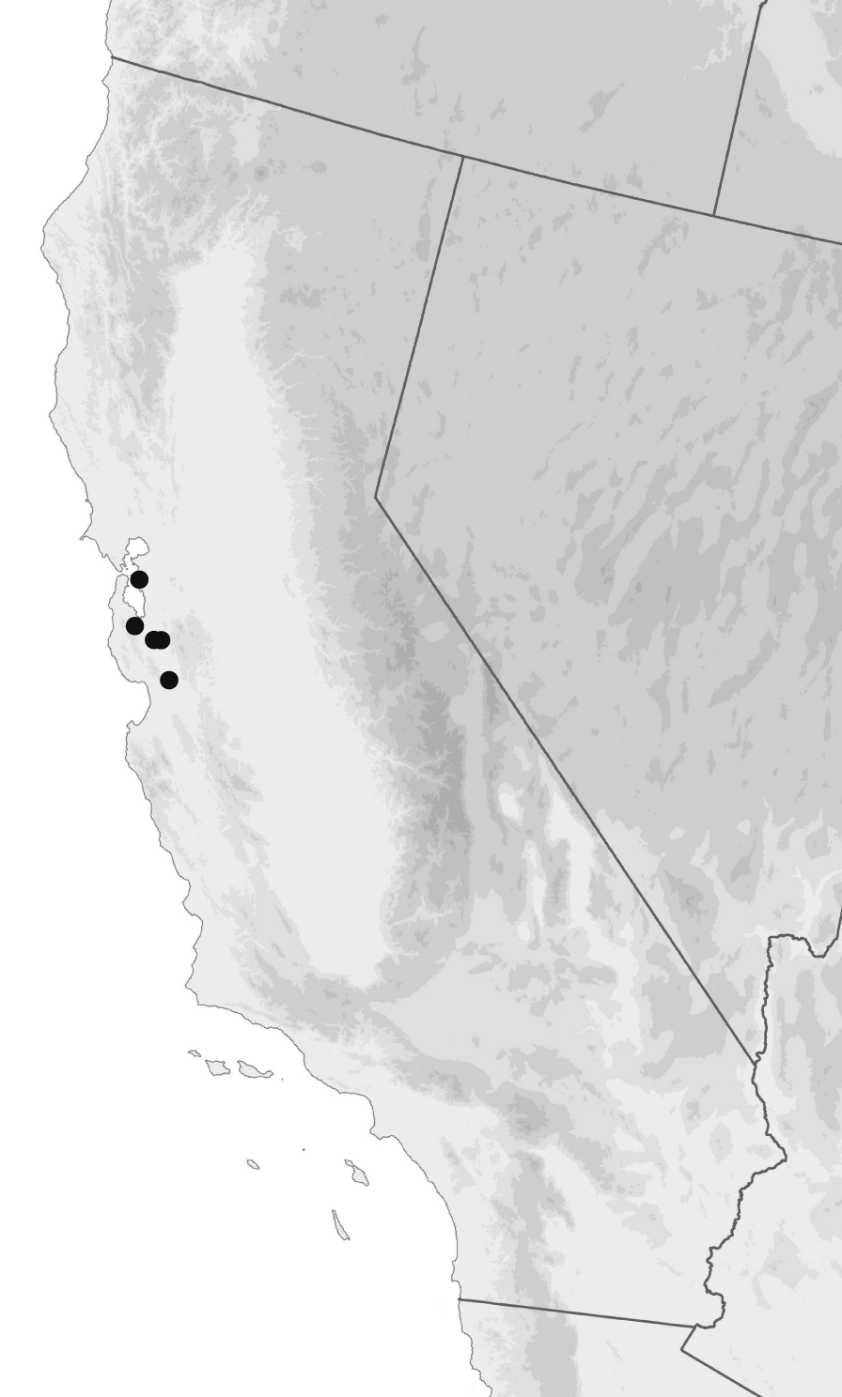
Geographic distribution of *Bembidion ambiguum* in California.

A Mediterranean species, from Spain and Portugal east to Israel and Iraq, on various Mediterranean islands, and in northern Africa from Morocco to Algeria, Tunisia, and Libya, and the Azores (Matallah et al. 2016, Ortuño and Toribio 2005, Marggi et al. 2017). In North America, it is has been found at five localities in the San Francisco Bay, California region (Fig. 5).

The records from California, from the earliest to latest records, are as follows:

- USA: California, Santa Clara Co., Palo Alto, Stanford University Campus, 37.4224°N 122.18124°W. 12.xi.2012. Lindgren funnel trap, Sirex lure, collector R. Sloan. (1 female, JRLC.)
- USA: California, Santa Clara Co., Santa Clara, Forge Garden and Santa Clara University, 37.35229°N 121.93941°W. 20.v.2013. (1 male, EMEC346362/1 female EMEC346363.)
- USA: California, Santa Clara Co., Gilroy, Thousand Trails, 4.xi.2015. Lindgren funnel trap with Quercivorol, collector R. Sloan. (1 female, JRLC.)
- USA: California, Santa Clara Co., San Jose, 1890 Dobbin Drive, 21.xii.2016. Lindgren funnel trap with Manuka oil, collector C. Higgenbottom. (1 male, JRLC.)
- USA: California: Alameda Co., Oakland, 37.8120°N 122.2717°W. 23.iv.2017, 19.xi.2017, and 2.xii.2017. Collector S. Crews. (1 male, EMEC346364/1 female EMEC346365; 1 male, OSAC DRMaddison DNA Voucher DNA5191.)

#### Ecology

In Oakland, California, the beetles were found in a backyard in an urban environment with non-native grasses and a mixture of native and non-native shrubs. Collections were made during the day, and the specimens were found in leaf litter around a small coffeeberry (*Frangula californica*), under a birdbath on moist soil, and at the base of dandelions (*Taraxacum officinale*).

#### Taxon discussion

The key of Lindroth (1963) to species groups of *Bembidion* can be modified as follows to accommodate the *B. ambiguum* species group (page 209):

7. 8. elytral stria removed from lateral bead at least half as much as from 7. stria ................. 8

– 8. stria closer to side-margin ................. 7a

7a. Anterior supraorbital seta at the front end of a deep furrow; this furrow is separated from the center of the head by a carina ................. *ambiguum* group

– Anterior supraorbital setae not in furrow, without carina mesal to this ................. 10

The key of Lindroth (1963) to species of *Bembidion* can be similarly modified as follows to accommodate *B. ambiguum* (page 213):

20. 8. elytral stria removed from lateral bead (at middle) at least half as much as from 7. stria. (All striae evident to apex) ................. 21

– 8. stria much closer to side-margin ................. 20a

20a. Anterior supraorbital seta at the front end of a deep furrow. Pronotum slightly constricted at base. Metallic coloration; with strong isodiametric microsculpture over the entire dorsal surface. Elytra with discal setae in shallow foveae. 3.5–4.2 mm in length ................. *B. ambiguum*

– Anterior supraorbital seta not in deep furrow. Remaining characters not as above ................. 31

## Discussion

It has been suggested that introduced species establish only 10% of the time or less (Williamson and Fitter 1996), and that establishment or the failure to establish is a complex interaction between the life history traits of the introduced species and climate, competition, predation, disease, and disturbance regime (Sakai et al. 2001). Prior to introduction potential propagules must be transported to a novel area. The primary route for transport and initial introductions of carabid beetles into North America has been hypothesized as via ship ballast dirt and with imported plant materials (see summary in Sokolov and Kavanaugh (2014)). Ship ballast would only apply to very old introductions, as this practice is no longer used and was never typical in the San Francisco Bay area. Sokolov and Kavanaugh (2014) suggest that importation of nursery stock is also very limited in the San Francisco region. Thus, the limited extent in California of the two primary routes for initial transport may account for the small number of introduced carabids established in that region of North America. Only 13 species are reported as introduced carabid species in California (Bousquet 2012); of those, nine are established and restricted to the north central region of California centered on San Francisco. The remaining four are only known from a small number of records and are either restricted to the far north of the state or are probably not established. This pattern contrasts with what is found in northwestern North America where Spence and Spence (1988) found 21 introduced carabid species in British Columbia and up to 17 introduced species in areas near Vancouver, BC. In addition to a potentially low rate of introductions, the characteristic and unique climatic regime of the Mediterranean-type ecosystem, with mild, relatively wet winters and warm, dry summers and frequent extended periods of drought, may be a significant barrier to the numerous north-temperate-climate adapted species that have successfully established via adventive introduction elsewhere. Although this may account for the absence of some species, why are Mediterranean climate adapted species like *B. ambiguum* not establishing more frequently given that they are not climate-limited in California? We speculate that this is due to the rarity of direct transport of plant material, especially nursery stock from other Mediterranean regions.

The earliest known record in for *B. ambiguum* in North America is from 2012. Given the span of its distribution at this time, it is likely well-established. In addition to being adapted to California's climate, *B. ambiguum* appears to be tolerant of disturbance, can take advantage of urban and agricultural mesic microhabitats, and can withstand high-levels of toxicity in their environment (Cárdenas and Hidalgo 2006). It is likely this species will continue to spread in the region.

## Supplementary Material

XML Treatment for Bembidion (Neja) ambiguum

## Figures and Tables

**Figure 4a.**
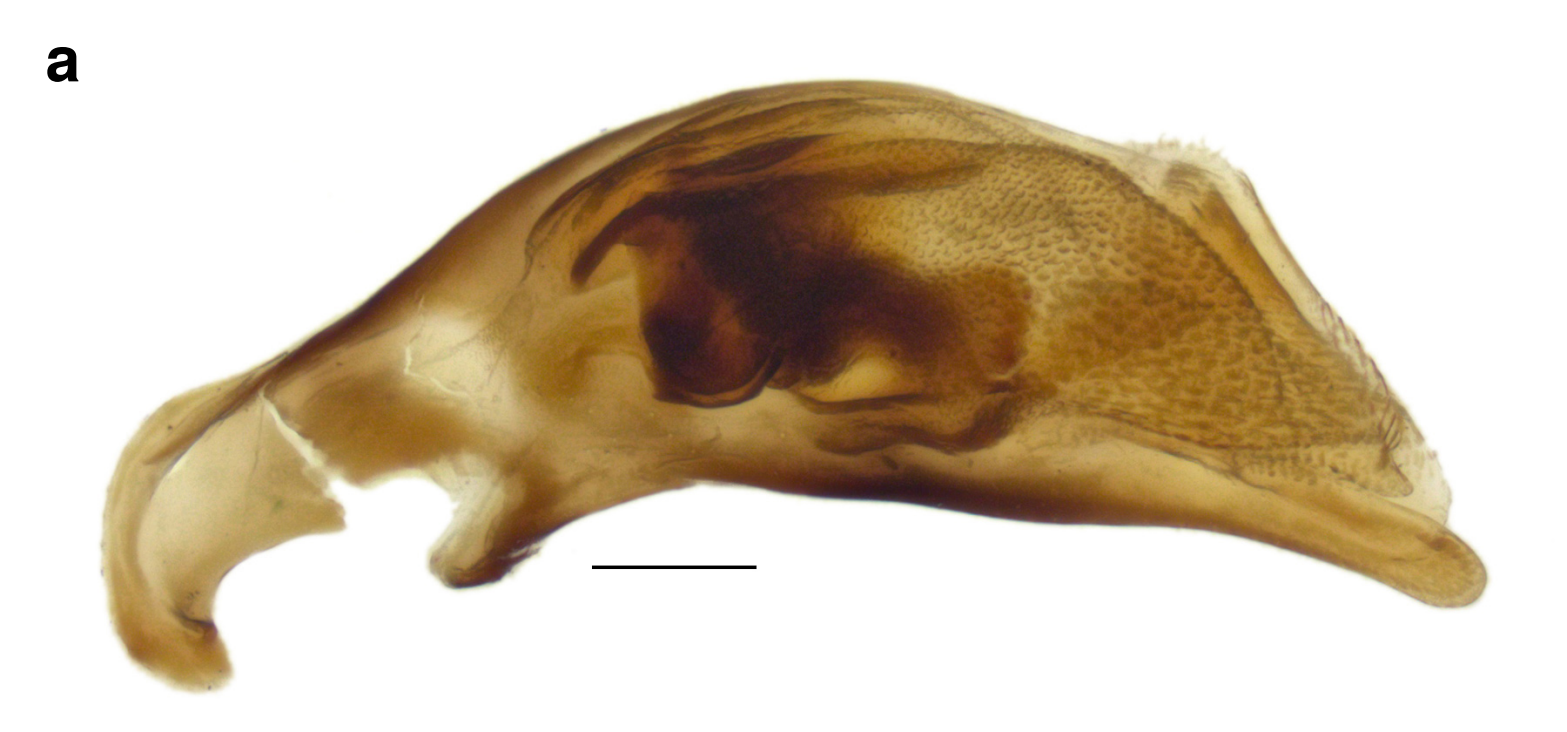
Specimen from Oakland, California, USA.

**Figure 4b.**
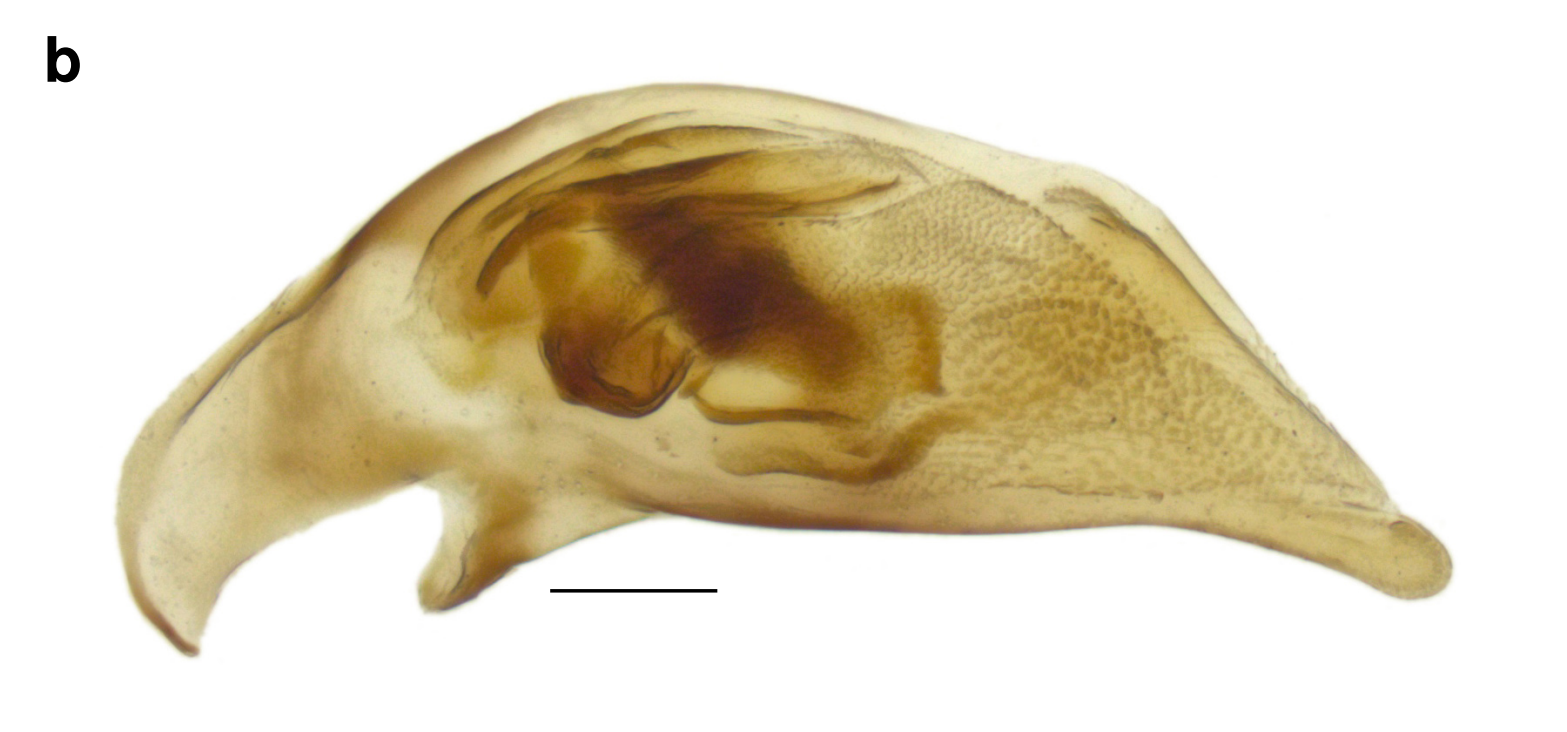
Specimen from 20 km SE Nador, Kariat-Arkmane, Morocco
